# Severe hypoglycemia reduces the shivering threshold in rabbits

**DOI:** 10.1186/s12871-019-0794-7

**Published:** 2019-07-09

**Authors:** Keiichi Wada, Taishi Masamune, Hirofumi Ino, Kenta Ueda, Tadahiko Ishiyama, Daniel I. Sessler, Takashi Matsukawa

**Affiliations:** 1 Surgical Center, University of Yamanashi Hospital, 1110 Shimokato, Chuo, Yamanashi, 409-3898 Japan; 2 Department of Anesthesia, Yamanashi Prefectural Central Hospital, Kofu, Japan; 3 Department of Anesthesiology, University of Yamanashi, Chuo, Japan; 4 Department of Outcomes Research, Anesthesiology Institute, Clinic, Cleveland, USA

**Keywords:** Hypoglycemia, Shivering, Rabbits

## Abstract

**Background:**

We previously reported that each 100 mg dL^− 1^ reduction in blood glucose over the range from ≈90 to > 300 mg dL^− 1^ decreases the shivering threshold (triggering core temperature) in rabbits by 1 °C. However, the effects of lower blood glucose concentrations has yet to be evaluated. We thus evaluated the relationship between the shivering threshold and blood glucose concentration over the mild-to-severe hypoglycemic range.

**Methods:**

Thirty-nine rabbits were lightly anaesthetized with isoflurane and randomly assigned to one of the three groups: 1) severe hypoglycemia, insulin and dextrose infusions titrated to achieve blood glucose concentration at 45–75 mg dL^− 1^; 2) mild hypoglycemia, insulin and dextrose infusions titrated to achieve blood glucose concentration at 75–100 mg dL^− 1^; and 3) saline infusion. Cooling by colonic perfusion of water at 10 °C was continued until shivering occurred or esophageal core temperatures reached to 34 °C.

**Results:**

The shivering threshold in the severe hypoglycemic rabbits was 35.7 ± 1.1 °C (mean ± SD); the thresholds in the mild hypoglycemic rabbits was 37.0 ± 0.7 °C; and the threshold in the control rabbits was 37.9 ± 1.0 °C. The shivering threshold increased linearly with blood glucose concentration: shivering threshold (°C) = 0.032 ∙ [blood glucose concentration (mg dL^− 1^)] + 34.1, R^2^ = 0.45. The shivering threshold thus decreased by approximately 1 °C for each 31 mg dL^− 1^ decrease in blood glucose concentration.

**Conclusions:**

There was a linear relationship between blood glucose and the shivering threshold over the range from severe hypoglycemia to normoglycemia. Blood glucose perturbations in the hypoglycemic range reduced the shivering threshold about three times as much as previously reported for the hyperglycemic range.

## Background

The initial thermoregulatory defence against cold exposure is arterio-venous shunt vasoconstriction [1]. Although restricted largely to fingers and toes, shunt vasoconstriction constrains metabolic heat to the core thermal compartment and thus helps maintain core temperature [2]. Shivering is a delayed response and does not occur until core temperature is about 1 °C below the threshold (triggering core temperature) for vasoconstriction [3]. Thus by the time humans start to shiver, they are thus already somewhat hypothermic. Shivering is an effective defence that can acutely augment metabolic rate by about a factor-of-five [4]. Humans can maintain a factor-of-three elevation in metabolic heat production for 3–4 h before muscles tire [5]. In adult humans, nonshivering thermogenesis has a limited role [6, 7].

Accidental hypothermia can result from extreme cold exposure, but more commonly results from mild environmental exposure combined with regulatory failure [8]. Regulatory failure is often consequent to drug exposure, especially alcohol. Nevertheless, ethanol has relatively little direct effect on the threshold for vasoconstriction [9], and the effect of ethanol on shivering remains unclear. Intoxication may contribute to hypothermia because it is often associated with hypoglycaemia [10] which may also contribute to “hiker’s hypothermia” [11].

Hypoglycemia impairs cold defenses by exciting warm-sensitive neurons and inhibiting cold-sensitive neurons [12]. Hypoglycemia inhibits cold responses in humans, and qualitatively contributes to hypothermia [10, 13–15]. Consistent with these observations, we have previously demonstrated a substantial and linear increase in the shivering threshold over the normal-to-hyperglycemic range [16]. However, the effect of moderate-to-severe hypoglycemia on the shivering thresholds remains unknown. We thus quantified the effect of hypoglycemia on the shivering threshold in rabbits.

## Methods

Forty male Japanese white rabbits, obtained from KITAYAMA LABES CO.,LTD., roughly 20–30-week-old (mean weight, 3.2 kg; range, 3.0–3.6 kg), fasted for 24 h were used with the approval of the Committee on Animal Research at the Faculty of Medicine, University of Yamanashi. Their core temperatures in the daytime are approximately 39 °C. The ambient temperature in the laboratory was kept about 26 °C with an air conditioner. To eliminate the effect of circadian variation on body temperature, we commenced the study at approximately 10:00 and typically concluded at approximately 16:00.

### Protocol

Animals were prepared as our previous studies [17, 18]. Briefly, we anaesthetized a rabbit by inhalation of 4% isoflurane and 67% nitrous oxide with oxygen. Then, we performed tracheal intubation with a 4.0-mm inner diameter endotracheal tube after administration of local anesthesia by 8% lidocaine spray. After that, the rabbit was permitted to continue spontaneous breathing.

An intravenous catheter was then inserted in a marginal ear vein to infuse lactated Ringer’s solution at 3–4 mL kg^− 1^ h^− 1^. We also inserted another catheter in a femoral artery for invasive blood pressure measurement, blood gas analysis and blood glucose determination. Body temperature was maintained to prevent hypothermia by water perfusion at a temperature of approximately 40 °C via a plastic tube positioned in the colon.

Rabbits were randomly assigned without stratification to one of the three groups by a computer-generated randomization list: 1) severe hypoglycemia, insulin and dextrose infusions were titrated to achieve blood glucose concentrations between 45 and 75 mg dL^− 1^; 2) mild hypoglycemia, insulin and dextrose infusions were titrated to achieve blood glucose concentrations between 75 and 100 mg dL^− 1^; and, 3) control, infusion of saline. The randomization list was created at once when this study was planned, and it was accessed by one investigator when he prepared the drug explained above. The allocation was concealed except the investigator until the last practical time. There were 13 rabbits each in the severe and mild hypoglycemia groups, and 14 in the control group.

In both hypoglycemic groups, insulin (1 U mL^− 1^) was infused at 3 U kg^− 1^ h^− 1^ starting shortly after induction of anesthesia. The insulin infusion rate was adjusted or dextrose 20% was infused as necessary to produce blood glucose concentrations within designated target ranges. Blood glucose concentrations were stable between 120 and 150 min after the initiation of infusion. The end-tidal concentration of isoflurane was then reduced to 0.2 minimum alveolar concentration, specifically approximately 0.4%, with nitrous oxide discontinued in order for the animal to breathe in 100% oxygen.

Hypothermia was induced by perfusion of water at 10 °C through a plastic tube in the colon about 30 to 60 min after the stabilization of hypoglycemia. Water flow was adjusted as necessary (maximum 3 L min^− 1^) to reduce core temperature at a rate of 2–3 °C h^− 1^. The study concluded when shivering occurred or a core temperature of 34 °C was reached. At the end of the experiment, the rabbit was euthanized by means of administration of intravenous potassium chloride under deep anesthetization as requested by the Committee on Animal Research.

### Measurements

Arterial blood was sampled for blood glucose concentration (Nipro Stat Strip XP; Nipro, Osaka, Japan) at least hourly and at the shivering threshold in each rabbit. Blood gas analysis and blood glucose were obtained from an i-STAT (Abbott Point of Care Inc., Princeton, NJ). According to the manufacturer, i-STAT blood gas and glucose values are highly correlated with those from a Radiometer ABL Flex (correlation coefficient = 0.91–1.00) over the entire clinical range, typically not differing by more than 5 mg/dl.

Anesthesia monitors (Infinity Delta; Draeger Medical Systems, Danvers, MA) with a thermometer and an anesthetic gas analyzer (Scio; Drägerwerk AG & Co. KGaA, Lübeck, Germany) provided electrocardiograms, arterial blood pressure, body temperature, and end-tidal gas concentrations. All data were captured electronically.

Shivering was defined as systemic vigorous muscular contraction accompanied by erection of the hair of the skin. The shivering threshold was defined as the core temperature when shivering was initially observed. Shivering was evaluated via visual inspection by an observer blinded to the treatment and blood glucose concentration. Core temperatures were obtained and recorded from a thermometer placed in the distal esophagus. The thermometer we used was accurate to ±0.1 °C at temperatures ranging from 25 to 45 °C. Arterial blood was sampled at the time when shivering was first observed or when the study concluded at a temperature of 34 °C.

After induction of general anesthesia as above, the rabbit’s head was shaved using animal clippers to position purpose-built electroencephalogram electrodes (BIS sensor XP small size; Aspect Medical Systems, Norwood, MA), which were connected to a bispectral index monitor (A-2000; Aspect Medical Systems, Norwood, MA) in order to assess the degree of sedation using the method of Shibuya and colleagues [19].

### Statistical analyses

A sample-size estimate indicated that 13 rabbits per group were sufficient to detect a 0.95 °C difference in esophageal temperature between groups with an α = 0.05 and a power of 0.8 on the basis of a standard deviation (SD) of 0.7 °C. We were able to find no studies previously reported significant clinical or physiological differences in the esophageal temperature at which shivering occurs in response to moderate-to-severe hypoglycemia. Thus, the study was powered to detect a difference in the esophageal temperatures between groups of 0.95 °C according to results of preliminary experiments and our previous study.

Data were analyzed by one-way analysis of variance with Tukey–Kramer’s test. The relationship between individual blood glucose concentrations and corresponding shivering thresholds were evaluated by linear regression analysis. Statistical analyses were performed using Excel 2007 (Microsoft Corporation, Redmond, WA) with the add-in software Statcel 3 [20]. Results are presented as means ± SDs. *P* < 0.05 was considered statistically significant.

## Results

One rabbit in the control group was excluded because of severe hyperglycemia, to approximately 300 mg dL^− 1^. Two rabbits in the severe hypoglycemic group did not shiver at the time when the core temperature reached to 34 °C, accordingly they were treated as if they had shivered at 34 °C as in our previous study [18]. Characteristic data prior to cooling for each group are shown in Table 1. Except for blood glucose concentration, no substantive differences in characteristics prior to cooling were observed among the groups.

**Table 1:** Characteristics before cooling

	Severe hypoglycemia	Mild hypoglycemia	Control
Tested (n)	13	13	13
Heart rate (beats min^− 1^)	283 ± 24	287 ± 17	280 ± 17
MAP (mmHg)	72 ± 14	79 ± 9	81 ± 14
Respiratory rate (breaths min^− 1^)	71 ± 11	67 ± 10	71 ± 12
BIS value	84 ± 15	73 ± 17	71 ± 17
Esophageal temperature (°C)	39.0 ± 0.7	39.1 ± 0.8	39.5 ± 1.0
Arterial pH	7.45 ± 0.07	7.46 ± 0.05	7.49 ± 0.07
PaCO2 (mmHg)	30.5 ± 4.9	28.7 ± 5.4	27.9 ± 5.1
PaO2 (mmHg)	426 ± 137	439 ± 72	479 ± 88
Base excess (mmol L^− 1^)	− 2.9 ± 4.0	− 3.5 ± 3.4	− 1.9 ± 4.5
K+ (mmol L^− 1^)	2.9 ± 0.4	2.9 ± 0.2	3.0 ± 0.3
Lactate (mmol L^− 1^)	4.1 ± 1.4	4.3 ± 2.0	4.7 ± 2.1
Glucose (mg dL^− 1^)	64 ± 10^*†^	85 ± 7^*^	120 ± 21

Data are expressed as means ± SDs MAP: mean arterial blood pressure

*P < 0.05; compared with the control group

†*P* < 0.05; compared with the mild hypoglycemia group

The measured values at the time of shivering are shown in Table 2. Some differences in hemodynamic and respiratory values were observed among the groups at the time when shivering occurred. Hypnotic levels, as assessed by BIS, were comparable in each group.

**Table 2:** At the shivering threshold

	Severe hypoglycemia	Mild hypoglycemia	Control
Shivered (#/n)	11/13	13/13	13/13
Heart rate (beats min^−1^)	227 ± 26^*†^	263 ± 22	274 ± 18
MAP (mmHg)	71 ± 13^*^	85 ± 21	88 ± 19
Respiratory rate (breaths min^− 1^)	63 ± 12	63 ± 15	59 ± 13
BIS value	86 ± 10	87 ± 8	79 ± 12
Esophageal temperature (°C)	35.7 ± 1.1^*†^	37.0 ± 0.7	37.9 ± 1.0
Arterial pH	7.40 ± 0.05^*^	7.42 ± 0.04	7.47 ± 0.06
PaCO2 (mmHg)	35.3 ± 5.8^*^	32.5 ± 4.0	30.3 ± 5.2
PaO2 (mmHg)	516 ± 120	490 ± 48	514 ± 65
Base excess (mmol L^− 1^)	2.7 ± 3.2	3.5 ± 3.0	− 1.4 ± 4.6
K+ (mmol L^− 1^)	2.6 ± 0.3	2.7 ± 0.3	2.9 ± 0.3
Lactate (mmol L^− 1^)	3.3 ± 1.1	3.7 ± 1.7	3.9 ± 1.3
Glucose (mg dL^− 1^)	62 ± 14^*†^	83 ± 11^*^	114 ± 18

Data are expressed as means ± SDs

MAP mean arterial blood pressure

*P < 0.05; compared with the control group

†*P* < 0.05; compared with the mild hypoglycemia group

**Fig. 1:**
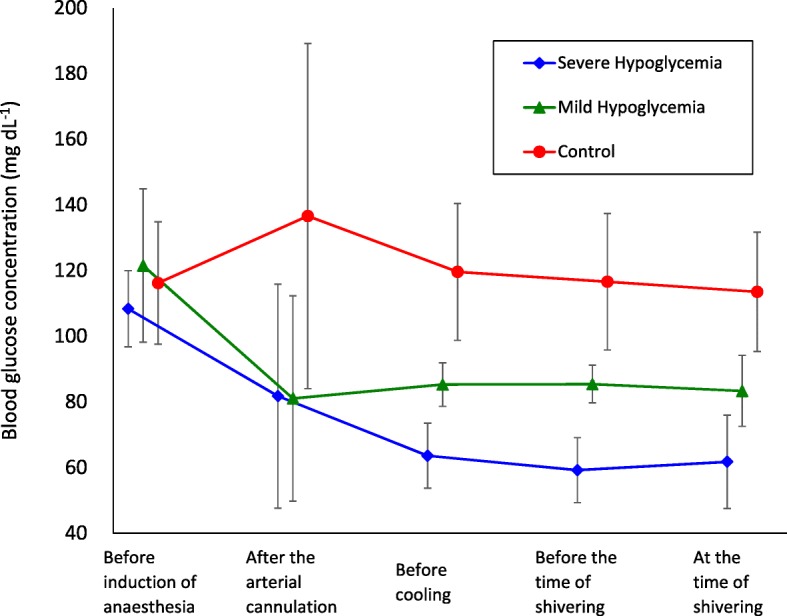
The average blood glucose concentration at each point of time in each group. Diamonds, severe hypoglycemia group; triangles, mild hypoglycemia group; filled circles, control group

**Fig. 2:**
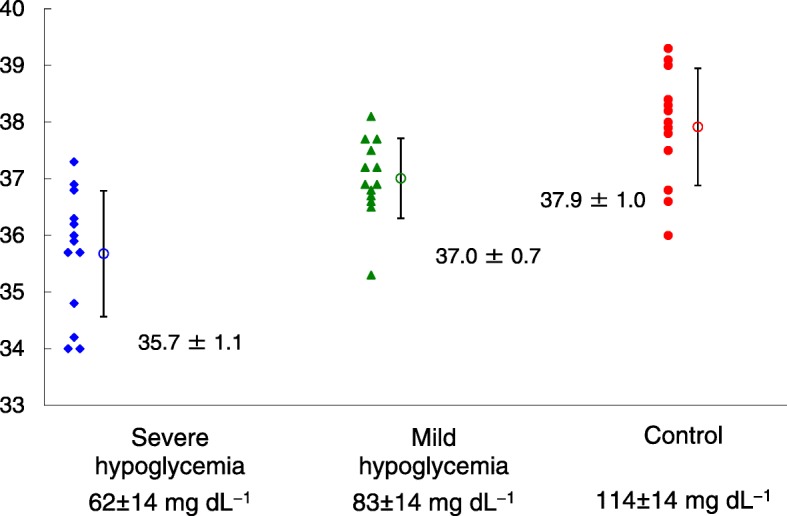
Shivering thresholds in each group. Diamonds, severe hypoglycemia group; triangles, mild hypoglycemia group; filled circles, control group. Open circles with SD error bars represent the mean shivering threshold in each group. *P < 0.05

Figure 1 shows blood glucose concentrations over time in each group. In the severe hypoglycemic group, the mean blood glucose concentration was 62 mg dL^− 1^ with shivering thresholds of 35.7 °C. In the mild hypoglycemic group, it was 83 mg dL^− 1^ with 37.0 °C. In the control group, it was 114 mg dL^− 1^ with 37.9 °C (Table 2). Figure 2 shows individual and average temperatures for each group. A significant difference in the shivering threshold was observed between the severe hypoglycemic group and the mild hypoglycemic group, and between the severe hypoglycemic group and the control group.

**Fig. 3:**
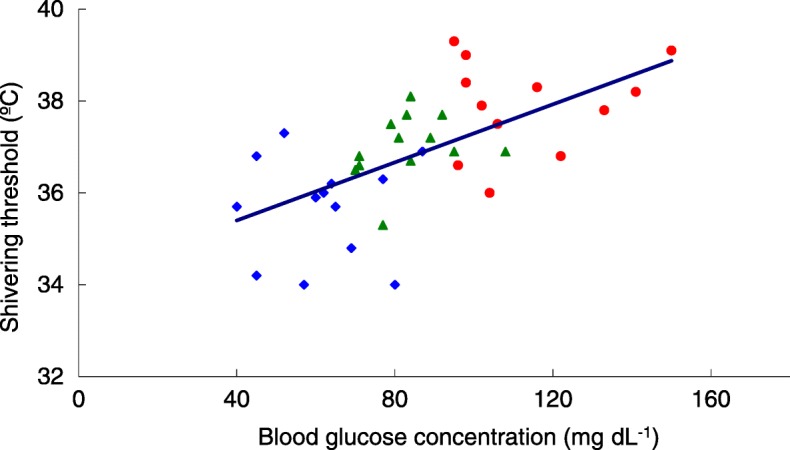
Linear regression model of individual blood glucose concentrations versus individual shivering thresholds. Diamonds, severe hypoglycemia group; triangles, mild hypoglycemia group; filled circles, control group. Decreased blood glucose concentrations were associated with decreased shivering thresholds. Shivering threshold (°C) = 0.032 ∙ [blood glucose (mg dL^− 1^)] + 34.1; R^2^ = 0.45. The shivering threshold thus decreased by approximately 1 °C for each 31 mg dL^− 1^ decrease in blood glucose concentration

Figure 3 shows individual blood glucose concentrations at the time of shivering versus core temperature (i.e., the shivering threshold). The shivering threshold linearly increased as a function of blood glucose concentration: shivering threshold (°C) = 0.032 ∙ [blood glucose (mg dL^− 1^)] + 34.1; the coefficient of determination (R [2]) = 0.45. The shivering threshold thus decreased by approximately 1 °C for each 31 mg dL^− 1^ decrease in blood glucose concentration.

## Discussion

The shivering threshold decreased by approximately 1 °C for each 31 mg dL^− 1^ decrease in blood glucose concentration over the hypoglycemic range. This result differs markedly from our previous study which showed that the shivering threshold increased by approximately 1 °C for each 100 mg dL^− 1^ increase in blood glucose concentration in the hyperglycemic range. Moderate-to-severe hypoglycemia thus reduces the shivering threshold about three times more than a comparable change in the hyperglycemic range. Consequently, the shivering threshold decreased more than 2 °C when blood glucose was reduced from 114 ± 18 mg dL^− 1^ to 62 ± 14 mg dL^− 1^, a highly clinically important amount. Common causes of hypoglycemia include alcohol intoxication and strenuous exercise, especially with inadequate food intake. Our results suggest that accidental hypothermia may be caused or aggravated by hypoglycemia.

The normal blood glucose concentration in New Zealand white rabbit is thought to be 122 ± 15 mg dL^− 1^ (range from 81 to 183 mg dL^− 1^) [21]. The normal blood glucose range for Japanese white rabbits might thus be roughly 100–140 mg dL^− 1^. Blood glucose concentrations less than 80 mg dL^− 1^ therefore constitute distinct hypoglycemia. We therefore defined severe hypoglycemia as blood glucose concentrations 45–75 mg dL^− 1^, and mild hypoglycemia as at 75–100 mg dL^− 1^. It is therefore likely that the blood glucose concentration in the control group was within the normal blood glucose concentrations in rabbits.

We used both dextrose and insulin to regulate the blood glucose concentrations. To produce appropriate blood glucose concentrations, we needed more insulin than in our previous study [16]. Thus, we cannot exclude the possibility that insulin per se reduced the shivering threshold — although it seems unlikely that insulin per se directly affects shivering, rather than via its effect on blood glucose.

Heart rates at the shivering threshold were about 15% lower in the severely hypoglycemic group than in the other two groups. Hypoglycemia per se can provoke bradycardia [22, 23]. But hypothermia does as well [24] and is a more likely explanation for the small observed differences.

We defined shivering as systemic vigorous muscular contraction. Normal shivering in humans is thought to be characterized by 4–8 cycle min^− 1^ systemic intensity variation [25], however, we were unable to quantify shivering patterns since we did not record electromyograms. We are thus unable to exclude the possibility of mistaking non-thermoregulatory movement for shivering, although sustained volitional movement during anesthesia would be most unusual.

In this study, there are some limitations. First, because of restriction of the equipment in our laboratory, the rabbits were ventilated with 100% oxygen which resulted in high PaO2. This may have influenced the results. Second, as mentioned above, we cannot completely rule out the possibility of deeming rabbits that did not shiver as shivering. Last, we did not research neuromuscular abnormalities responsible for the observed changes. We wish to take into consideration these factors in our further studies.

## Conclusions

In summary, hypoglycemia linearly and substantially reduced the shivering threshold in rabbits, and the reduction was three-fold greater in this range than at hyperglycemic concentrations. For example, the shivering threshold decreased more than 2 °C when blood glucose was reduced from 114 ± 18 mg dL^− 1^ to 62 ± 14 mg dL^− 1^, a highly clinically important amount. Our results thus suggest that moderate-to-severe hypoglycemia impairs cold defenses and is likely to promote accidental hypothermia.

## Data Availability

The datasets used and/or analysed during the current study are available from the corresponding author on reasonable request. Please contact us: kwada@yamanashi.ac.jp.

## Abbreviation

SD: standard deviation
